# Evaluation of hepatitis B knowledge, practices, and beliefs among the Jordanian population: A cross-sectional study

**DOI:** 10.1371/journal.pone.0277186

**Published:** 2022-11-04

**Authors:** Bayan Othman, Muna Barakat, Amin Omar, Amani Al-Rawashdeh, Yazan Qashou, Rafat Zrieq, Mohammad A. A. Al-Najjar

**Affiliations:** 1 Department of Pharmaceutical Sciences and Pharmaceutics, Applied Science Private University, Amman, Jordan; 2 Department of Clinical Pharmacy & Therapeutics, Applied Science Private University, Amman, Jordan; 3 Faculty of Pharmacy, Amman Arab University, Amman, Jordan; 4 Department of Public Health, College of Public Health and Health Informatics, University of Ha’il, Ha’il, Saudi Arabia; Shahid Beheshti University of Medical Sciences, School of Dentistry, ISLAMIC REPUBLIC OF IRAN

## Abstract

This study aimed to assess the knowledge, practices, and beliefs among the Jordanian population regarding hepatitis B virus (HBV) infection. A cross-sectional questionnaire was designed and used to recruit participants from October 5th through December 12^th^. Statistical analysis was conducted using SPSS. Descriptive statistical analysis was used to analyse the sociodemographic data, the Shapiro-Wilk test was used to assess the normality, Cronbach’s α was used to evaluate the reliability of the questionnaire and Point-biserial correlation was used to figure out whether there is an association between Score of knowledge and the dichotomous variables. A random sample of 432 participated in the study. The majority were females (n = 310, 71.8%), the mean age was 21 (42.0%) years,416 (96.3%) were urban inhabitants and most of them (n = 351, 81.3%) had bachelor’s degree. School/university (n = 280, 64.8%) were reported as a major source of information followed by TV/internet/social media 276 (63.9%). The total mean (± SD) of knowledge score regarding HBV infection symptoms, transmission modes and treatment was found 12.28 ± 3.2. Participants’ knowledge regarding symptoms including nausea, vomiting and loss of appetite was 73 (16.9%). More than 80% had good knowledge regarding the complications of HBV infection. Only 100 participant reported vaccination (23.1%) against the virus. Poor knowledge and low vaccination rate against HBV were found thus implementing comprehensive educational program for people highlighting the importance of vaccination against the virus is crucial.

## Introduction

Hepatitis B virus (HBV) is a serious viral infection that attacks the liver causing acute and chronic disease conditions and ultimately may causes cirrhosis and hepatocellular carcinoma. HBV can be transmitted through percutaneous or mucosal exposure to infected blood and various body fluids. Moreover, the virus can be transmitted from mother to child during child birth [1]. According to the World Health Organization (WHO) estimations in 2019 that 296 million people were living with chronic HBV infection, while 820,000 were reported global deaths due to cirrhosis and hepatocellular carcinoma complications [2].

In the WHO’s Eastern Mediterranean Region, it is estimated that 60 million people are infected with HBV [2]. Several hepatitis prevalence studies were conducted in Middle Eastern countries including Jordan [3–5]. In Jordan estimated population of the Kingdom by Administrative Divisions at end-year of 2021 was reported to be eleven million fifty-seven thousand [6]. The national prevalence of HBV in Jordan is estimated to be around 2.4% according to the published Polaris observatory modelling study in 2016 [7, 8]. In 2015 the Jordanian National Blood Bank recorded a 2% of the unceasing screening for HBV infection among blood donors [9]. However, recent studies reported unexplained increment in the prevalence rates of HBV and hepatitis C virus (HCV) infections among blood donors in 2019 and among pregnant females in Jordan (5%) [5, 10]. To add on, a recent cluster of new cases of acute hepatitis of unknown origin among young children in multi-countries were reported by the WHO [11].

As new cases of HBV infection are still being reported till this day progressively, according to WHO, vaccination is a major strategy for the elimination of HBV infection [12]. Since 1995, Jordan’s Ministry of Health has made vaccination against HBV mandatory for all new-borns in an effort to stop the spread of the virus.

Improving the knowledge of symptoms and causes towards HBV infection among people are crucial strategies to eliminate the transmission of the disease [13]. Therefore, several studies have been conducted worldwide to assess people’s knowledge and beliefs towards HBV its infection [14–16]. In Jordan, unfortunately, the assessment of HBV knowledge, practice and beliefs have never been explored among the general population. Therefore, there is a need to assess the knowledge, practices, and beliefs of the Jordanian people towards HBV infection. This study was intended to supplement our previous findings [10] in order to design a based program for the society.

## Materials and methods

### Study design and settings

An electronic-based cross-sectional survey was used to recruit eligible participants in this study. Eligible participants were approached from northern, center, and southern governorates in Jordan. The study questionnaire was designed using a Web-based survey software (Google Forms). From October 5th through December 12^th^, online social media was used to approach the participants and collect data for this study. All eligible participants were requested to provide their written consent before study participation. Participants who agreed to participate were requested to answer the questionnaire anonymously to ensure confidentiality.

### Survey development

The questionnaire (S1 Appendix) was adopted and modified based on similar studies and the general principles of good survey design [17]. To ensure comprehension and clarity the questionnaire was validated by experienced colleagues (n = 2) in the clinical Pharmacy & Therapeutics research field. It was translated from English to Arabic and refinements were made as needed. The survey contained close-ended questions that can be completed within an average of 15 minutes.

The questionnaire consisted of 4 parts. The first part was designed to collect participant demographic characteristics including gender, age, governorates, educational level, occupation, income, smoking and marital status. The second part was designed to evaluate participants’ knowledge of HBV infection including symptoms, transmission modes and treatment. The third part was designed to evaluate practices related to HBV infection and vaccination against it. The last part was designed to assess participants’ beliefs about HBV and people infected with the virus. The questionnaire included closed-ended questions consisted of a Likert scale (Strongly agree, Agree, Undecided, Disagree, strongly Disagree) and (Yes/No) assessments. Knowledge score is a number that was given to each individual and it is calculated by giving a 1 for each correct answer and a 0 for each incorrect answer. The scale measured knowledge from a maximum of 22 to a minimum of Zero. Scores < 13 were considered poor, ≥ 13 as adequate knowledge of Hepatitis B [18].

### Sample size

Based on the number of estimated population of the Kingdom by Administrative Divisions (6) the sample size was calculated using a margin of error of 5%, a confidence level of 95% and a response distribution of 50% giving a minimum sample size of 384 [19].

### Study sample

A convenience sampling method was conducted and a total of 432 applicants participated in the study. Inclusion criteria included individuals aging 18 years and above, with no mental illnesses and residents in Jordan. Patients with mental illnesses and visitors from other countries were excluded from data collection.

### Statistical analysis

Data were exported from Google forms to Microsoft Excel and analysed using the Statistical Package for the Social Sciences (SPSS), Version 25.0 (SPSS Inc., Chicago, IL, USA). Descriptive statistical analysis was used to analyse the sociodemographic data: medians and interquartile ranges (IQR) for continuous variables. Categorical variables were demonstrated as frequencies and percentages. The Shapiro-Wilk test was used to assess the normality. Cronbach’s α was used to evaluate the reliability of the questionnaire, i.e., that the scales constructed are fit for their purpose, with values ≥ 0.7 indicating acceptable internal consistency. Point-biserial correlation was used to figure out whether there is an association between Score of knowledge and the dichotomous variables presenting the Pearson correlation and P-values.

### Ethics statement

Ethical approval was obtained from the Institutional Research Board in the faculty of Pharmacy Applied Science Private University (Approval number: 2021-PHA-42).

## Results

### Demographic characteristics

A total of 432 applicants participated in this study and the principal characteristics of the sample are outlined in Table 1. The majority of respondents were females (n = 310, 71.8%) compared to males (n = 122, 28.2%). The study participants’ mean age was 21 (42.0%) years and 416 (96.3%) were urban inhabitants. The educational level was bachelor’s degree for 351 (81.3%) participant and 352 (81.5%) were non-smokers and unemployed. About half of the study participants (n = 220, 50.9%) had a monthly income of less than 250 Jordanian Dinar (JD). In addition, most of the participants were single (n = 381, 88.2%).

**Table 1:** Sociodemographic characteristics of the participants (n = 432).

Parameter	Median (IQR)	n (%)
**Age (years)**	21.0 (42.0)	
**Gender**		
Female		310 (71.8)
Male		122 (28.2)
**Governate**		
North of Jordan		16 (3.7)
Middle (including the capital of Jordan)		394 (91.2)
South of Jordan		22 (5.1)
**Residential area**		
Urban		416 (96.3)
Rural		16 (3.7)
**Educational level**		
Illiterate		0 (0.0)
Primary education		0 (0.0)
secondary education		32 (7.4)
Diploma		32 (7.4)
Bachelor’s degree		351 (81.3)
Higher education		17 (3.9)
**Occupation**		
Employed in health sector		33 (7.6)
Employed in non-health sector		47 (10.9)
Unemployed or students		352 (81.5)
**Monthly income**		
Less than 250 JD		220 (50.9)
251–500 JD		88 (20.4)
501–750 JD		34 (7.9)
751–1000 JD		38 (8.8)
More than 1000 JD		52 (12.0)
**Marital status**		
Single		381 (88.2)
Married		46 (10.6)
Widow or divorced		5 (1.2)
**Smoking status**		
Smoker		67 (15.5)
Ex-smoker		13 (3.0)
Non-smoker		352 (81.5)

**Fig 1:**
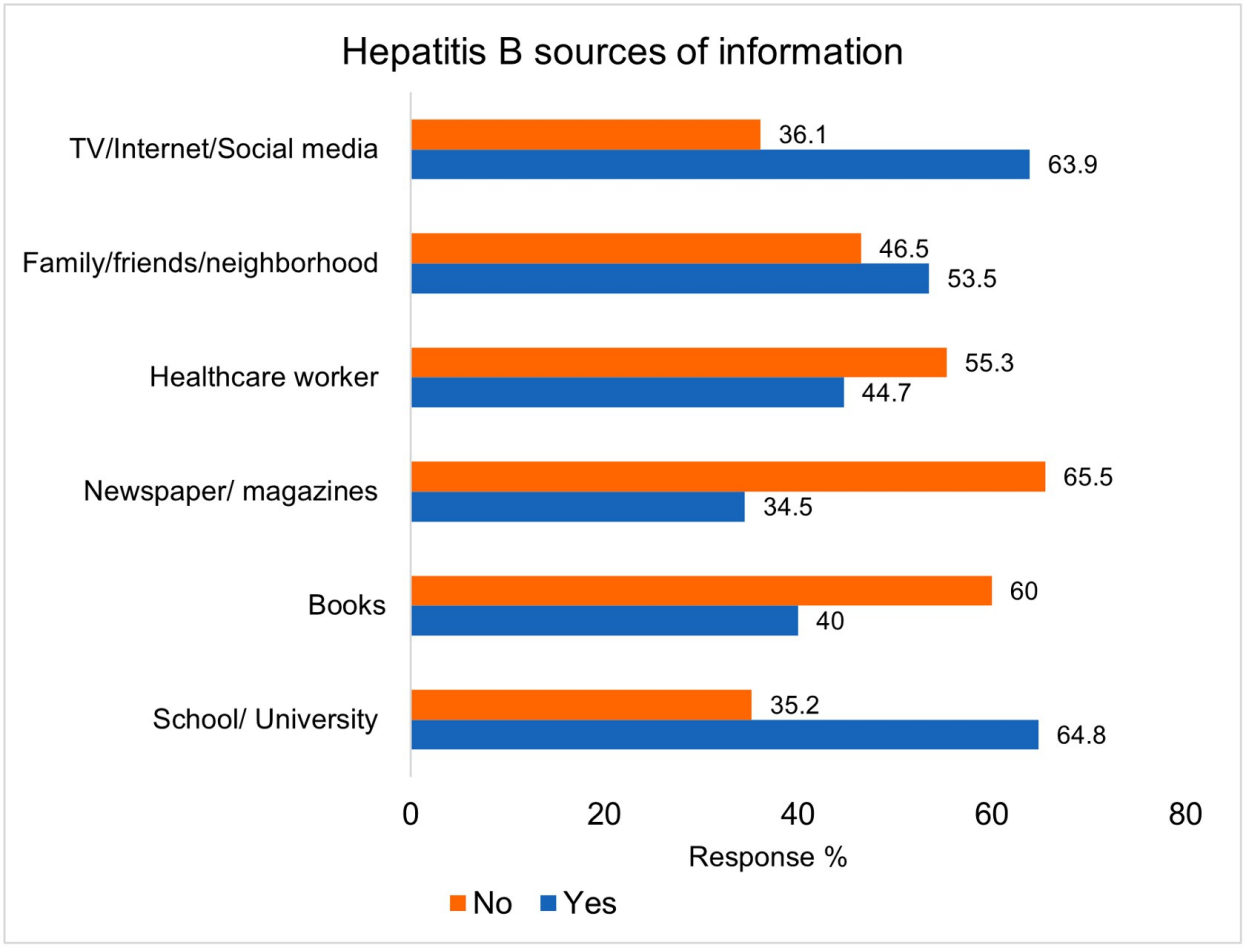
Participants sources of information on hepatitis B (n = 432).

As for the participants reported sources of information regarding hepatitis B, more than half (n = 280, 64.8%) reported school/university as a major source of information followed by 276 (63.9%) by TV/internet/social media as shown in Fig 1.

### Evaluation of participants’ knowledge about HBV infection

The responses of the participants towards HBV infection’s knowledge questions are described in Table 2. Knowledge was assessed by questions focusing on the aetiology, signs and symptoms, transmission, treatment and management of HBV infection. The questionnaire scale ranged between 22 (maximum) and 0 (minimum). A score level of < 13 was considered poor while a score of ≥ 13 was considered adequate knowledge score regarding HBV infection. The mean (±SD) knowledge score of study participants was found 12.28 ± 3.2.

**Table 2:** Participants’ knowledge about hepatitis B (n = 432).

Statement	Correct Answer*	
	n	%
**Is hepatitis B a viral disease?**	269	62.3
**Can hepatitis B affect any age group?**	321	74.3
**Are all of the following among the common symptoms of hepatitis B?**		
• Cold and flu (fever, running nose, cough)	279	64.6
• Jaundice (the skin, whites of the eyes and mucous membranes turn yellow)	383	88.7
• Nausea, vomiting and loss of appetite	73	16.9
**Hepatitis B can be transmitted by**		
• Un-sterilized syringes, needles and surgical instruments	362	83.8
• Contaminated blood and blood products	376	87.0
• Contaminated blades of the barber/ear and nose piercing	301	69.7
• Unsafe sexual intercourse	275	63.7
• Transmitted from mother to foetus	302	69.9
• Contaminated water/food prepared by person suffering with these infections	110	25.5
**Is Hepatitis B curable?**	29	6.7
**Can hepatitis B be self-cured by body without medical treatment?**	37	8.6
**Is there a specific diet required for the treatment of hepatitis B?**	24	5.6
**People who are infected with hepatitis B put others at risk of getting infected.**	254	58.8
**Patients with Hepatitis B infection should be restrained from sexual contact.**	196	45.4
**Is each of the following considered a complication of hepatitis B?**		
• Affect liver function	418	96.8
• Liver cancer	303	70.1
• Liver cirrhosis	382	88.4
• Death	350	81
**Is vaccination available against hepatitis B?**	196	45.4
**How many doses of the hepatitis B vaccine should be given?**	66	15.3

*Knowledge was assessed by giving 1 to the correct Answer and 0 to the Wrong Answer. The scale measured knowledge from a maximum of 22 to a minimum of Zero. Scores < 13 were considered poor, ≥ 13 as adequate knowledge of Hepatitis B. Mean knowledge score was 12.28 ± 3.2.

Out of the total number of participants, 269 (62.3%) knew that HBV infection is a viral disease. Poor knowledge was evident in responses to questions of common symptoms of HBV infection, amongst nausea, vomiting and loss of appetite (n = 73,16.9%). Only 29 (6.7%), 37 (8.6%) and 24 (5.67%) knew that HBV infection is curable, can be self-cured by the body without medical treatment and that there is no specific diet for HBV patients, respectively. Moreover, approximately half of the participants (n = 196, 45.5%) knew the availability of a vaccine against the virus while only 66 (15.3%) of all study participants knew that there were three doses of the vaccine to be taken. On the contrary, more than 80% had good knowledge regarding complications of HBV infection. The majority (n = 383, 88.7%) knew that jaundice is one of the most common symptoms of HBV infection.

### Evaluation of practices regarding HBV infection

As shown in Table 3, practices toward HBV infection were measured by 6 questions. Each response was answered with a ’yes’ or ’no’. Most of the respondents, (n = 388, 89.8%), never went for HBV screening and 429 (99.3%) had never been infected with hepatitis B. The majority of participants (n = 404, 93.5%) reported their willingness to go for further investigation and treatment if they were diagnosed with HBV. On the other hand, 141 participants (32.6%) reported that lack of information about the test is the possible reason for not being tested for HBV. Regarding vaccination, 100 respondents (23.1%) only reported vaccination against HBV; on the other hand, most participants (n = 306, 70.8%) reported fear of the side effects caused by HBV vaccine as the major reason for not being vaccinated.

**Table 3:** Participants practice regarding hepatitis (n = 432).

Question	Yes	No
**Have you ever been screened for hepatitis B?**	44 (10.2)	388 (89.8)
**Have you ever been infected with hepatitis B?**	3 (0.7)	429 (99.3)
**If you were diagnosed with hepatitis B, would you go for further investigation and treatment?**	404 (93.5)	28 (6.5)
**From your perspective, the possible reasons against being tested for hepatitis B could be**		
• Insufficient money	48 (11.1)	384 (88.9)
• lack of information about the test	141 (32.6)	291 (67.4)
• Lack of time	109 (25.2)	323 (74.8)
• Fear of positive test result	66 (15.3)	366 (84.7)
• Other	137 (31.7)	295 (68.3)
**Have you got yourself vaccinated against hepatitis B?**	100 (23.1)	332 (76.9)
**Reasons for not being vaccinated.**		
• Insufficient money	49 (11.3)	383 (88.7)
• I don’t know where to be vaccinated.	140 (32.4)	292 (67.6)
• Lack of time	94 (21.8)	338 (78.2)
• Lack of sufficient information	29 (6.7)	403 (93.3)
• Fear of side effects	306 (70.8)	126 (29.2)
• Others	152 (35.2)	280 (64.8)

### Participant’s beliefs regarding HBV infection

**Fig 2:**
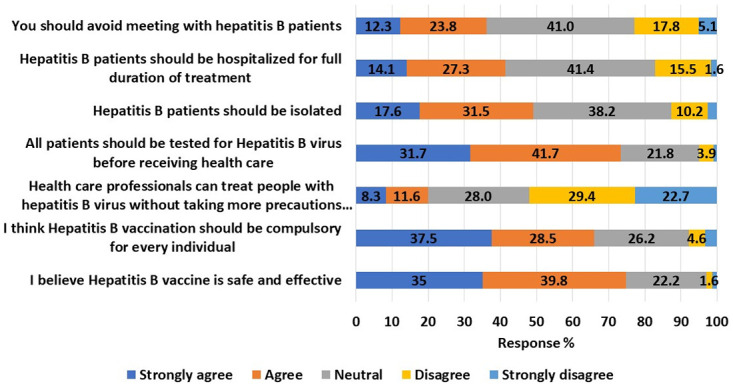
Participants’ beliefs about hepatitis B and people infected with the virus (n = 432).

Fig 2 revealed that 12.3% (n = 53) of participants reported that they should avoid meeting with HBV patients and 14.1% (n = 61) and 17.6% (n = 76) thought HBV patients should be hospitalized for the full duration of treatment and should be isolated, respectively. Conversely, 31.7% (n = 137) of participants thought all patients should be evaluated for HBV before receiving health care. Moreover, only 37.5% (n = 162) and 35% (n = 151) of the study respondents believed that vaccination against HBV should be compulsory for every individual and the vaccine is safe and effective, respectively.

Point-biserial correlation between Score of knowledge about hepatitis B and the dichotomous demographic variables (Table 4) showed that knowledge score was correlated significantly with female gender and educational level. While the other variables were not statistically correlated with knowledge level.

**Table 4:** Point-biserial correlation between Score of knowledge about hepatitis B and the dichotomous demographic variables.

Variables	First Dose-Score of symptoms	
	Pearson Correlation	P-value*
• **Age**: <21 vs >21	0.018	0.695
• **Gender:** Female vs Male	**0.091**	**0.025**
• **Marital status**: Unmarried vs married	0.046	0.311
• **Residential area**: Urban vs rural	0.025	0.667
• **Educational level**: University degree vs non-University degree	**0.074**	**0.034**
• **Employment status**: employed vs non-employed	-0.032	0.073
• **Monthly income**: >250 vs <250 JD	-0.032	0.083
• **Smoking status**: smoker vs Non-smoker	-0.051	0.268

*Significance measure at P-value<0.05 is presented in bold using Point-biserial correlation

## Discussion

HBV infection is a major serious global health problem that threatens the life of millions of people annually. Therefore, intensive efforts are done by the governments to combat the spreading of this chronic disease. In addition to the compulsory vaccination program, people should be equipped with knowledge and awareness concerning this infectious disease. This study investigates the knowledge, practices, and beliefs of the Jordanian general population about HBV infection. Although most of the participants in the study have tertiary education and above, their general knowledge regarding HBV infection was poor, and 17% of them had never heard about HBV infection before. Their correct answers about the ways of being infected with HBV were satisfactory. In contrast, most of their answers were incorrect regarding the knowledge about the treatment and whether the disease is curable. However, the participants showed positive perceptions about the vaccine and the vaccination process.

It is well documented that socioeconomic indicators such as monthly income and the level of education are strongly associated with knowledge about health [20, 21]. Our results suggested a statistical positive correlation between the educational level and the knowledge level about HBV, on the other hand, there was no correlated with monthly income, knowing that the monthly income of for more than half of the participants was ≤250JD which is considered below the poverty line according to Jordan’s official statistics [22]. A previous study conducted in Jordan among pregnant females reported that the participants knowledge score was inversely correlated to their socioeconomic status [10]. In addition, a Malaysian study revealed that members with higher education and high family income had better knowledge about HBV infection [23]. Similarly, knowledge about HBV was positively associated with the participant’s educational levels in Mauritania, Somaliland [24, 25]. Other studies have reported similar findings, leading to limited knowledge and awareness of HBV infection among pregnant females [26–28]. In the context of Jordan, although the educational system in Jordan has been extensively accessed and encouraged by the government and the people themselves, the outcomes of this study represent the unexpectedly low satisfactory level of knowledge among the public. Similarly, a recent study conducted in Jordan among medical students reported lack of knowledge regarding HIV/AIDS [29]. Such findings regrading infectious diseases reveal a noteworthy gap in the efforts devoted to educating people regarding infectious diseases such as HBV/HIV through seminars, health campaigns on social media and TV, and other activities. Italy, for example, started a workshop program to educate the people about HBV infection in 2008 [30] and the following series of workshops were established ten years later [31]. The epidemiological changes of HBV infection among blood donors over a 10-year period in Italy was assessed. Significant reduction in the rates of HBV infection among first time and repeat donors was reported. The prevalence of HBV markers in first time donors was 175.6×105, significantly (p<0.01) decreasing over time from 275.9×105 in 2009 to 143.6×105 in 2018. Also, the rates of HBV new infections were 2.68×105 decreasing (p<0.05) from 3.37×105 in 2009 to 2.17×105 in 2018 [32].

The very high percentage of the participants who mentioned that they wouldn’t go to test for HBV may originate from their fear of discovering the infection and its management complication later [33]. Another possible reason could be due to there is not enough knowledge about the infection, most participants might think it is not essential to care about it. Normally, vaccination is a successful defence mechanism against HBV infection, which significantly reduces the rates of HBV infection as reported [34].

Here, it is worth mentioning the Ministry of Health in Jordan initiated a mandatory vaccination program for new-borns against HBV in 1995 [35]. Although most of the study participants were young (mean = 21 years old), which suppose that most if not all of them were subjected to the compulsory vaccination upon delivery, only fifth reported vaccination against HBV, which might be forced to take it because of their careers’ requirements or because they were considered a high-risk group. From their point of view, a strong justification for not taking the vaccine reported was the fear vaccination side effects. Similarly, Froerhlich and West showed that 55% of their study population had a serious fear of side effects of the HBV vaccine [36]. Indeed, this is a global issue that might result from the lack of enough information or from the conspiracy that human beings are targeted, which was spread more during the COVID-19 pandemic [37–39].

## Limitations

Several limitations can be identified in this study. First, an online Google survey is subject to a security breach, yet password protection for editing privileges was implemented and accessible by the research team. Second, the representation of the Jordanian population could be compromised, as the study tool warrants computer literacy, internet availability, an enhanced level of education to access and complete the online survey which might affect the generalizability of our study findings. Third, information bias related to the accessibility of resources on-demand can compromise response credibility. Fourth, selection bias related to the snowball collection technique might be an issue, with no random selection warranted. Residual confounding bias could arise from possible un-measured variables or responses to variables directly or indirectly related to stroke. Moreover, an online survey instead of a face-to-face meeting poses reliability and authenticity risks to the study data. The online survey included country-specific questions for Jordanians to complete, with a full description of the target population and inclusion criteria in the title and the invitation message. Considering the restriction measures during the COVID-19 pandemic, such a methodology was the best option.

## Conclusion

Overall, the results of this study call for the need for a comprehensive educating program containing important information about HBV infection. The program should include information about the virus, ways of infection, complications, treatment, and the importance of vaccination. The program can be conducted via workshops, social media, and campaigns in the shopping malls and other communication ways. A testing campaign should also accompany this program for HBV, which should cover all governorates in Jordan.

## Supporting information

S1 Appendix Evaluation of hepatitis B knowledge, practices, and beliefs among the Jordanian population: A cross-sectional study (English version). (DOCX)

S2 Appendix Evaluation of hepatitis B knowledge, practices, and beliefs among the Jordanian population: A cross-sectional study (Arabic Version). (DOCX)
