# Supplementary material for: Evaluation of hepatitis B knowledge, practices, and beliefs among the Jordanian population: A cross-sectional study
Source: PLoS One. 2022 Nov 4;17(11):e0277186. doi: 10.1371/journal.pone.0277186 (PMC9635692; doi:10.1371/journal.pone.0277186)
Supplement: S1 Appendix — (DOCX) [file pone.0277186.s001.docx]

**Appendix 1. Evaluation of hepatitis B knowledge, practices, and beliefs among the Jordanian population: A cross-sectional study**

This questionnaire was prepared by a group of researchers at the Faculty of Pharmacy at the Applied Science Private University to conduct an assessment study of the extent of knowledge of hepatitis B, beliefs and to assess the vaccination status against it among the population of Jordan. Therefore, we ask your honorable person to answer this questionnaire accurately and clearly, knowing that the data contained in this questionnaire is prepared for purely academic purposes, and accordingly we pledge to maintain the strict confidentiality of all the data contained in it and not to use it for anything other than the purpose for which it was written.

**Your participation in filling out the questionnaire is highly appreciated**

**I agree to participate**

**I do not agree to participate**

**Section 1: Sociodemographic characteristics**

**Gender**

Male

Female

**Age**: ……………… (fill in years please)

**Governate**

Northern governates (Irbid, Ajloun, Jerash, Al-Mafraq)

Center governates (Amman, Al-Zarqa, Al-Balqa, Madaba)

Southern governates (Al-Karak, Al-Tafila, Ma’an, Aqaba)

**Residence area**

Urban

Rural

**Educational level**

Illiterate

Primary education

secondary education

Diploma

Bachelor’s degree

Higher education

**Major**

Health related major (Medical doctor, dentist, pharmacist, nurse, nutritionist ….)

Other

**Occupation**

Employed in health sector

Employed in non-health sector

Unemployed

**Income**

Less than 250 JD

251-500 JD

501-750 JD

751-1000 JD

More than 1000 JD

**Marital status**

Single

Married

Widow or divorced

**Smoking status**

Smoker

Ex-smoker

Non-smoker

**Section 2: Participant’s knowledge of hepatitis B**

**Have you ever heard of hepatitis B?**

Yes

No

**If the previous question answer is yes from where? (You can choose more than one answer)**

|  | **Yes** | **No** |
| --- | --- | --- |
| School/ University |  |  |
| Books |  |  |
| Newspaper/ magazines |  |  |
| Healthcare worker |  |  |
| Family/friends/neighborhood |  |  |
| Tv/internet/social media |  |  |

**Is hepatitis B a viral disease?**

Yes

No

I don’t know

**Can hepatitis B affect any age group?**

Yes

No

I don’t know

**Are all the following among the common symptoms of hepatitis B?**

|  | Yes | No |
| --- | --- | --- |
| Cold and flu (fever, running nose, cough) |  |  |
| Jaundice (the skin, whites of the eyes and mucous membranes turn yellow) |  |  |
| Nausea, vomiting and loss of appetite |  |  |

**Hepatitis B can be transmitted by**

|  | **Yes** | **No** |
| --- | --- | --- |
| un-sterilized syringes, needles and surgical instruments |  |  |
| Contaminated blood and blood products |  |  |
| Contaminated blades of the barber/ear and nose piercing |  |  |
| Unsafe sexual intercourse |  |  |
| Transmitted from mother to fetus |  |  |
| Contaminated water/food prepared by person suffering with these infections |  |  |

**Is Hepatitis B curable/treatable?**

Yes

No

I don’t know

**Can hepatitis B be self-cured by body without medical treatment?**

Yes

No

I don’t know

**Is there a specific diet required for the treatment of hepatitis B?**

Yes

No

I don’t know

**People who are infected with hepatitis B put others at risk of getting infected.**

Yes

No

I don’t know

**Patients with Hepatitis B infection should be restrained from sexual contact.**

Yes

No

I don’t know

**Is each of the following considered a complication of hepatitis B?**

|  | **Yes** | **No** |
| --- | --- | --- |
| Affect liver function |  |  |
| Liver cancer |  |  |
| Liver cirrhosis |  |  |
| Death |  |  |

**Is vaccination available against hepatitis B?**

Yes

No

I don’t know

**How many doses of the hepatitis B vaccine should be given?**

1

2

3

I don’t know

**Section 3. Practice related to hepatitis B.**

**Have you ever been screened for hepatitis B?**

Yes

No

**Have you ever been infected with hepatitis B?**

Yes

No

**If you were diagnosed with hepatitis B, would you go for further investigation and treatment?**

Yes

No

**What are your reasons for not being tested?**

|  | **Yes** | **No** |
| --- | --- | --- |
| I don’t have enough money |  |  |
| I don’t know where to test |  |  |
| I don’t have time |  |  |
| Fear of positive test result |  |  |
| Other |  |  |

**Have you got vaccine against hepatitis B?**

Yes

No

**Reasons for not being vaccinated.**

|  | **Yes** | **No** |
| --- | --- | --- |
| I don’t have enough money. |  |  |
| I don’t know where to be vaccinated. |  |  |
| I don’t have time. |  |  |
| Fear of side effects |  |  |
| I don’t have sufficient information about the vaccine |  |  |
| Others |  |  |

**Section 4:** **Participants' beliefs about hepatitis B and people infected with the virus.**

**Please choose the answer that best expresses your belief in the following statements**

| Statement | Strongly agree | Agree | Neutral | Disagree | Strongly disagree |
| --- | --- | --- | --- | --- | --- |
| I believe Hepatitis B vaccine is safe and effective |  |  |  |  |  |
| I think Hepatitis B vaccination should be compulsory for every individual |  |  |  |  |  |
| Health care professionals can treat people with hepatitis B virus without taking more precautions than other patients |  |  |  |  |  |
| All patients should be tested for Hepatitis B virus before receiving health care |  |  |  |  |  |
| Hepatitis B patients should be isolated |  |  |  |  |  |
| Hepatitis B patients should be hospitalized for full duration of treatment |  |  |  |  |  |
| You should avoid meeting with hepatitis B patients |  |  |  |  |  |
