# Supplementary material for: Evaluation of hepatitis B knowledge, practices, and beliefs among the Jordanian population: A cross-sectional study
Source: PLoS One. 2022 Nov 4;17(11):e0277186. doi: 10.1371/journal.pone.0277186 (PMC9635692; doi:10.1371/journal.pone.0277186)
Supplement: S2 Appendix — (DOCX) [file pone.0277186.s002.docx]

**قياس مدى المعرفة و الوعي بالتهاب الكبد ب وتقييم حالة التطعيم بين السكان في الأردن**

**القسم أ: الخصائص الديموغرافية**

**الجنس**

ذكر

أنثى

**العمر**: ..................... (ملء في السنوات من فضلك)

**المحافظة**

محافظات الشمال ( إربد، عجلون، جرش، المفرق)

محافظات الوسط ( عمان، الزرقاء، البقعة ، مادبا)

محافظات الجنوب (الكرك، الطفيلة ،معان ،العقبة)

**مكان الإقامة**

مدينة

ريف

**المستوى التعليمي**

غير متعلم

تعليم ابتدائي

تعليم ثانوي

دبلوم

بكالوريوس

دراسات عليا

**التخصص الدراسي**

تخصص صحي ذو صلة بالقطاع الطبي (طبيب، طبيب أسنان، صيدلي، ممرض، أخصائي تغذية ....)

غير ذلك

**العمل**

أعمل حاليا

أعمل لحسابي الخاص

لا أعمل حاليا

**مستوى الدخل**

أقل من 250 دينار

251-500 دينار

501-750 دينار

751-1000 دينار

أكثر من 1000 دينار

**الحالة الاجتماعية**

اعزب

متزوج/ة

أرمل/ة أو مطلق/ة.

**حالة التدخين**

مدخن

مدخن سابق

غير مدخن

**هل سمعت عن التهاب الكبد ب؟**

نعم

لا

**إذا كانت إجابة السؤال السابقة نعم من أين؟ (يمكنك اختيار أكثر من إجابة واحدة)**

المدرسة / الجامعه

الكتب

الصحف / المجلات

موظف في القطاع الطبي (دكتور,صيدلاني, ممرض....)

العائلة/ الأصدقاء/الحي

التلفزيون/ الإنترنت/وسائل التواصل الاجتماعي

**هل سبق لك أن أصبت بالتهاب الكبد ب؟**

نعم

لا

لا أعلم

**هل سبق لك أن تم فحصك** لالتهاب **الكبد ب؟**

نعم

لا

لا أعلم

| **القسم ب: عناصر المعرفة حول طريقة أعراض انتقال العدوى، ومضاعفات فيروس التهاب الكبد ب.** | **نعم** | **لا** | **لا أعلم** |
| --- | --- | --- | --- |
| هل هناك عدة أنواع من التهاب الكبد؟ |  |  |  |
| هل التهاب الكبد ب مرض فيروسي؟ |  |  |  |
| هل يمكن أن يؤثر التهاب الكبد ب على أي فئة عمرية؟ |  |  |  |
| هل هناك أي أعراض ظاهرة لالتهاب الكبد ب في بعض المرضى؟ |  |  |  |
| يعتبر البرد والإنفلونزا (الحمى، سيلان الأنف، السعال) من بين الأعراض المبكرة لالتهاب الكبد ب؟ |  |  |  |
| هل يعد اليرقان ( اصفرار الجلد وابيضاض العينين) أحد الأعراض الشائعة لالتهاب الكبد ب؟ |  |  |  |
| هل الغثيان والقيء وفقدان الشهية من الأعراض الشائعة لالتهاب الكبد ب؟ |  |  |  |
| هل يمكن أن ينتقل التهاب الكبد ب عن طريق المحاقن والإبر والأدوات الجراحية غير المعقمة؟ |  |  |  |
| هل يمكن أن ينتقل التهاب الكبد ب عن طريق الدم الملوث و الأدوات الملوثه بالدم؟ |  |  |  |
| هل يمكن أن ينتقل التهاب الكبد ب باستخدام شفرات الحلاقة / ثقب الأذن والأنف ؟ |  |  |  |
| هل يمكن أن ينتقل التهاب الكبد ب عن طريق الجماع غير الآمن؟ |  |  |  |
| هل يمكن أن ينتقل التهاب الكبد ب من الأم إلى الجنين؟ |  |  |  |
| هل يمكن أن ينتقل التهاب الكبد ب عن طريق المياه/الطعام الملوث الذي يعده شخص مصاب بهذا المرض؟ |  |  |  |
| هل التهاب الكبد ب قابل للشفاء/العلاج؟ |  |  |  |
| هل يمكن علاج التهاب الكبد ب ذاتيا عن طريق الجسم؟ |  |  |  |
| هل التطعيم متاح ضد التهاب الكبد ب؟ |  |  |  |
| هل يوجد نظام غذائي محدد لعلاج التهاب الكبد ب؟ |  |  |  |
| هل يحتاج التهاب الكبد إلى مراقبة يومية؟ |  |  |  |
| هل تعرف كيف يتم إعطاء لقاح التهاب الكبد ب؟ |  |  |  |
| هل يعرض الأشخاص الحاملون لالتهاب الكبد ب الآخرين لخطر الإصابة ؟ |  |  |  |
| هل يمكن أن يؤثر التهاب الكبد ب على وظائف الكبد؟ |  |  |  |
| هل يمكن أن يسبب التهاب الكبد ب سرطان الكبد؟ |  |  |  |
| هل هناك أي علاقة بين التهاب الكبد وتليف الكبد؟ |  |  |  |
| هل يمكن أن يؤدي التهاب الكبد ب إلى الوفاة؟ |  |  |  |

| القسم ج: السلوكيات اتجاه التهاب الكبد من نوع ب | | | | | |
| --- | --- | --- | --- | --- | --- |
| السؤال | موافق بشدة | موافق | محايد | غير موافق | غير موافق بشدة |
| أعتقد أن لقاح التهاب الكبد ب آمن وفعال |  |  |  |  |  |
| أعتقد أن التطعيم ضد التهاب الكبد ب يجب أن يكون إلزاميا لكل فرد |  |  |  |  |  |
| المريض المصاب بعدوى التهاب الكبد ب يجب أن يمنع من الاتصال الجنسي مع شريكه |  |  |  |  |  |
| يجب على المتخصصين في الرعاية الصحية الامتناع عن علاج المرضى المصابين بالتهاب الكبد ب |  |  |  |  |  |
| يجب فحص جميع المرضى للكشف عن فيروس التهاب الكبد ب قبل تلقي الرعاية الصحية |  |  |  |  |  |
| ينبغي الإبلاغ عن كل حالة ثقب الجلد بالخطأ عن طريق الإبر |  |  |  |  |  |
| يجب عزل مرضى التهاب الكبد ب |  |  |  |  |  |
| يجب إدخال مرضى التهاب الكبد ب إلى المستشفى طوال فترة العلاج |  |  |  |  |  |
| طبيعة عملك يعرضك لخطر الإصابة بعدوى التهاب الكبد ب |  |  |  |  |  |
| نمط حياتك يعرضك لخطر الإصابة بعدوى التهاب الكبد ب |  |  |  |  |  |
| يجب أن تطلب حقنة جديدة قبل الاستخدام |  |  |  |  |  |
| يجب أن تطلب فحص الدم قبل عملية نقل الدم |  |  |  |  |  |
| يجب أن تطلب من الحلاق الخاص بك تغيير شفرة / أو معدات آمنة جديدة لثقب الأذن والأنف |  |  |  |  |  |
| في حال تم تشخيص إصابتك بالتهاب الكبد ب، سوف تذهب لإجراء المزيد من التحقيق والعلاج |  |  |  |  |  |
| يجب أن تتجنب مقابلة مرضى التهاب الكبد ب |  |  |  |  |  |
| لقد سبق وشاركت في برنامج تثقيف صحي متعلق بالتهاب الكبد ب |  |  |  |  |  |
| تشعر أنك بحاجة إلى مزيد من المعلومات حول التهاب الكبد ب |  |  |  |  |  |

| **القسم د : اختبار التهاب الكبد ب والتطعيم. . .** | | | |
| --- | --- | --- | --- |
| **سؤال** | **نعم** | **لا** | **لا أعرف** |
| هل حصلت على المطعوم المضاد لالتهاب الكبد ب؟ |  |  |  |
| هل تعتقد أنك تلقيت جدول تطعيم مكتمل؟ |  |  |  |

**أسباب عدم الفحص لالتهاب الكبد ب ؟**

ليس لدي أي سبب

ليس لدي ما يكفي من المال

لا أعرف أين أفحص لالتهاب الكبد ب

ليس لدي الوقت

الخوف من نتيجة الاختبار الإيجابية

**آخر.................... يرجى تحديد**

**أسباب عدم التطعيم**

ليس لدي أي سبب

ليس لدي ما يكفي من المال.

لا أعرف أين أفحص لالتهاب الكبد ب

ليس لدي الوقت

**آخر.................... يرجى تحديد**
